# An outbreak of Japanese encephalitis caused by genotype Ib Japanese encephalitis virus in China, 2018: A laboratory and field investigation

**DOI:** 10.1371/journal.pntd.0008312

**Published:** 2020-05-26

**Authors:** Wenjing Liu, Shihong Fu, Xuemin Ma, Xiaojing Chen, Dan Wu, Liwei Zhou, Qikai Yin, Fan Li, Ying He, Wenwen Lei, Yixing Li, Songtao Xu, Huaqing Wang, Zhenhai Wang, Huanyu Wang, Hong Yu, Guodong Liang

**Affiliations:** 1 Department of Pathogenic Biology, School of Basic Medicine, Qingdao University, Qingdao, People’s Republic of China; 2 Department of Arbovirus, NHC Key Laboratory of Biosafety, National Institute for Viral Disease Control and Prevention, Chinese Center for Disease Control and Prevention, Beijing, People’s Republic of China; 3 State Key Laboratory for Infectious Disease Prevention and Control, Chinese Center for Disease Control and Prevention, Beijing, People’s Republic of China; 4 Ningxia Hui Autonomous Region Center for Disease Control and Prevention, People’s Republic of China; 5 Department of Epidemiology, School of Medicine, Jinan University, Guangzhou, People’s Republic of China; 6 National Immunization Programme, Chinese Center for Disease Control and Prevention, Beijing, People’s Republic of China; 7 Center for Neurology, General Hospital of Ningxia Medical University, Ningxia, People’s Republic of China; CDC, UNITED STATES

## Abstract

Although Japanese encephalitis virus genotype Ib (JEV GIb) has replaced JEV GIII as the dominant genotype in endemic areas of Asia, no JEV GIb has been isolated from JE cases and natural mosquitoes at the same time in an outbreak of JE. In this study, we conducted virological and molecular biological laboratory tests on JE case samples (serum/cerebrospinal fluid) and locally collected mosquito samples from the 2018 JE outbreak in Ningxia, China. The result of JEV IgM antibody detection showed that 96% (67/70) of the suspected cases were laboratory-confirmed JE cases. Of the mosquitoes collected from local environments, 70% (17400/24900) were *Culex tritaeniorhynchus* of which 4.6% (16 /348 of the pools tested) were positive for JEV, other mosquitoes were negative. JEVs isolated from both the human cases and *C*. *tritaeniorhynchus* specimens belong to JEV GIb and are in the same evolutionary clade according to molecular evolution analyses.

JEV GIb was detected simultaneously from specimens of JE cases and mosquito samples collected in nature in this study, suggesting that the JE outbreak that occurred in Ningxia in 2018 was due to infection of JEV GIb.

## Author summary

Japanese encephalitis virus (JEV) is recognized as an important encephalitis pathogen all over the world. Its genotype is divided into GI-V. In recent years, JEV GIb (a temperate genotype) has gradually replaced GIII as the prevalent strain in JE endemic areas. Although JEV GIb originated from tropical Asia along with JEV GIa, it has rapidly spread for its advantages in wintering and infecting vectors. Although there have been epidemics caused by JEV GI and GIII, there have been no reports of a JE outbreak caused by JEV GIb alone in northeastern Asia. However, a JE outbreak occurred in the Ningxia Hui Autonomous Region in northern China in summer 2018 which was the first outbreak in Ningxia in recent decades. This paper presents a series of laboratory and field studies of this outbreak. The strain isolated from JE cases as well as JEV detected in *Culex tritaeniorhynchus* collected from local areas in nature all belonged to JEV GIb and were in the same evolutionary clade. This is the first report of a JE outbreak caused by JEV GIb infection in northeastern Asia (latitude 35 ° 14’– 39 ° 23’ N, longitude 104 ° 17’– 107 ° 39’ E), which used to be a low endemic area of JEV GIII.

## Introduction

Japanese encephalitis (JE) is a mosquito-borne arbovirus disease caused by Japanese encephalitis virus (JEV). JEV can circulate in several hosts: Aquatic wading birds are reservoir hosts [1,2], pigs are amplification hosts, and humans and equids are the terminal hosts. The first JEV was detected in Japan, which caused a pandemic that infected 6,000 people in 1924. Since then the virus has been found to be mosquito-borne. Humans can be infected with JEV through mosquito bites, particularly *Culex tritaeniorhynchus*, and clinical manifestations of fever and encephalitis may result [1]. Bioinformatics analyses have shown that JEV originated in Indonesia, Philippines and other places in southeast Asia, and has since spread to mainland Asia [3,4]. Although JEV is infectious to all age groups, JE mainly occurs in children and the mortality rate is approximately 30%. Although JE is a vaccine-preventable disease, it is prevalent in 24 countries and regions in Asia and Oceania. Approximately 3 billion people live in JEV endemic areas and are threatened by JEV infection; therefore, JE represents a significant disease burden with a fatality rate of 20–30% and neurological or psychiatric sequelae in 30–50% of survivors [2,5,6]. JE has become a public health issue of international concern [7].

JEV is a positive RNA virus, and the length of the entire genome is ~ 11 kb. One polyprotein is encoded by the single open reading frame (ORF) of the genome, which can be divided into three structural proteins (capsid [C], membrane [M], and envelope[E]) and seven nonstructural proteins (NS1, NS2A, NS2B, NS3, NS4A, NS4B, and NS5) [1]. Molecular genetic evolution analyses based on the nucleotide sequence of the whole JEV genome and the E gene alone both divide JEV into genotypes I–V (JEV GI–GV) [3,8,9]. Studies have indicated that JEV GI can be further divided into two sub-genotypes, JEV GIa and JEV GIb. The former only circulates in Cambodia (KF192509), Thailand (KF192510.), and Australia (EF434785), while the latter is mainly distributed in areas north of 23°N in Eurasia [4,10]. In recent years, JEV most isolates from mainland China, Japan, and the Republic of Korea belong to JEV GIb, although these areas used to be endemic areas of JEV GIII [4,10].

Although there have been reports of the isolation of JEV GIb from humans [11], pigs, and mosquitoes [12] and the results provide evidence for the existence of JEV GIb, to the best of our knowledge, no outbreaks caused by JEV GIb among humans have been reported in Asia. This study acquired JEV GIb from both cerebrospinal fluid (CSF) specimens from JE cases and mosquito specimens collected from the local environment during the 2018 outbreak of JE in Ningxia (Ningxia Hui Autonomous Region) in northern China. Evidence from both laboratory and field data confirmed that JEV GIb was the pathogen that caused this outbreak. This study is the first to confirm that JEV GIb can cause JE outbreak in humans.

## Materials and methods

### Ethics statement

Ethical approval for this study was obtained by the Chinese Center for Disease Control and Prevention, JE has been a nationally notifiable disease in China since 1951 so the collection of data from JE cases is part of continuing public health surveillance of an infectious disease and thus was exempt from review by an institutional review board. Written informed consent was obtained from all adult participants and guardians of adolescent participants, and the data were analyzed anonymously.

### Specimen collection

Serum and CSF samples from suspected JE cases in the acute phase were collected at the hospital [11,13]. Mosquito samples were collected by UV traps (Kungfu Dude Mosquito & Fly Trap, LTS-M02; Wuhan Ji Xing Medical Technology Co, Wuhan, China) from breeding environments that included public land in villages and pigpens with the permission of owners/residents from 7:00 pm to 6:00 am the next morning. The mosquitoes were kept at −20°C for 1 h for anesthetization and then morphologically classified on ice. Both human and mosquito specimens were transported to the laboratory on dry ice [13,14].

Pools of 50 mosquito specimens were homogenized in a 1.5mL Eppendorf (EP) tube with 1 mL 4°C pre-cooled minimum essential medium (MEM) (10-010-CVR; Corning, Inc, Corning, NY, USA) containing 5% Penicillin (1000units/mL)Streptomycin(100μg/mL)(PS)(15070–063, GIBCOTM, Invitrogen, USA). The EP tube was then placed in an oscillating instrument (Retsch Tissue Lyser, Cat. No. 85220; QIAGEN, Hilden, Germany) and oscillated at a frequency of 25 oscillations s^−1^ for 3 min. The oscillated EP tube was centrifuged at 13,000 rpm for 30 min at 4°C, and the supernatant was stored at −80°C until detection [14,15].

### Virus IgM antibody detection

Since West Nile virus (WNV) and JEV are both mosquito-borne virus that can cause viral encephalitis (West Nile viral encephalitis is more likely to occur in adults) [16], and there is significant antigenic cross-reaction between the two viruses. Therefore, in this study, IgM antibodies, neutralizing antibodies to JEV and WNV, and viral genes (qRT-PCR) of the two viruses in serum samples and cerebrospinal fluid samples collected from cases in Ningxia were tested in parallel. Two JEV IgM antibody detection kits were used to test serum and CSF samples: The Beixi Capture ELISA 24 Test ELISA kit (REVI-001M, Lot: 1807–1; Shanghai B&C Biotechnology Co., Ltd., Shanghai, China) and the JE Detect IgM Antibody Capture ELISA Kit (Mac-ELISA, 96 Test Kit, JEMS-1, Lot: XC1285; InBios International, Inc, Seattle, WA, USA). The West Nile Virus (WNV) IgM antibody was detected by the Capture Dx Select Enzyme-linked Immunosorbent Assay ELISA Kit (96 Test, EL0300M, Lot: 1990N; Focus, Seattle, WA, USA), which does not support CSF specimen testing. The experimental procedures and interpretation of results were carried out in accordance with the manufacturer’s instructions [13,16].

### Neutralizing antibody detection

Neutralizing antibodies of serum samples were detected by 90% plaque reduction neutralization test (PRNT90). The detection of neutralizing antibody against WNV is same as that for JEV, and the strain used for WNV neutralizing antibody detection is WNV XJ11129 strain [16].

The Serum samples were serially diluted two-fold beginning at a 1:5 dilution and ending at 1: 160, then mixed with an equal volume JEV P3 strain at 200 pfu/100 μL. Different virus dilutions (100 pfu/100 μL, 10 pfu/100 μL, and 1 pfu/100 μL) were used as controls. The mixed solution was incubated at 37°C and 5% CO_2_ for 1 h. The incubated mixed solution was then added to BHK-21 newborn hamster kidney cells [16, 17] grown to 90% confluence in a single layer, and incubated at 37°C and 5% CO_2_ for 1 h. Then the cells were supplemented with 4 mL 1.2% methylcellulose-MEM containing 2% fetal bovine serum (FBS) and incubated for 3–5 days at 37°C and 5% CO_2_ until significant viral plaques were observed. Finally, the cell layers were stained with crystal violet, the number of viral plaques was counted, and the neutralizing antibody titer was calculated [16,17].

### Virus isolation

The mosquito grinding supernatant was filter-sterilized and inoculated into a 24-well plate (Corning, Inc, NY, USA) with Vero cells (African green ape kidney cells) grown to a 90% confluent monolayer. For virus isolation, 70 μL mosquito supernatant was added per well, followed by incubation at 37°C for 1 h. Then the cells were supplemented with 1 mL per well MEM containing 2% FBS. The cells were cultured at 37°C and 5% CO_2_, and the cytopathic effect (CPE) was observed daily. Specimens with no CPE were used for blind transmission for three generations [14,15]. CSF samples were inoculated into Vero cells at 100 μL/well for virus isolation following the same procedure [11].

### Detection of JEV and WNV by quantitative real-time reverse-transcription (qRT)-PCR

RNA was extracted from all collected samples (serum, CSF, and mosquito grinding supernatant) using a Tianlong Nucleic Acid Automatic Extractor (model: Np968.c; Suzhou Tianlong Biotechnology Co., Ltd., Jiangsu, China) with the Tianlong Nucleic Acid Extraction Kit (EX-RNA/DNA virus, Suzhou Tianlong Biotechnology Co., Ltd, Suzhou, China). All operations were carried out in accordance with the manufacturer’s instructions [18]. The Ready-To-Go kit (GE Healthcare, Little Chalfont, Buckinghamshire, UK) and random primers (pdN6) (TaKaRa, Shiga, Japan) were used to prepare the cDNA library [13,18].

qRT-PCR detection of JEV and WNV was performed on extracted RNA using the Stratagene real-time PCR instrument (model MX300; Thermo Fisher Scientific, Waltham, MA, USA) and the AgPath-IDTM One-step RT-PCR Kit (AM1005, Thermo Fisher Scientific, USA). Universal primers and probes for JEV (detect JEV genotype I, III and V) [18] and specific primers and probes for WNV [19] were used. Then the positive samples detected by qRT-PCR with JEV universal primers were amplified using primers specific for JEV E gene. The PCR products were subjected to nucleotide sequence determination and JE virus phylogenetic analyses were used to confirm the JEV genotype.

### Phylogenetic analyses of the JEV gene sequence

The E gene was amplified by semi-nested PCR. cDNAs of CSF and mosquito grinding supernatant were added to a 25-μL reaction volume as the instructions of the GoTaq Green Master Mix Kit (Promega, Madison, WI, USA). The primers used for the first round of PCR were JEV-E-1F (TTCATAGAAGGAGCCAGTGGA) and JEV-E-1R (TCGTTTAAACTCGCGACTGA), and the primers used for the second round were JEV-E-1F and JEV-E-2R (TTTCCCGAAAAGTCCACATC) [11,18]. The C+PrM gene sequence was also amplified by semi-nested PCR according to the same procedure. The primers used for the first round of PCR were JE-C+PrM-1F (CGTTCTTCAAGTTTACAGCATTAGC) and JE-C+PrM-1R (CCYRTGTTYCTGCCAAGCATCCAMCC), and the primers used for the second round were JE-C+PrM-1F and JE-C+PrM-2R (CGYTTGGAATGYCTRGTCCG). The product of the first round of PCR was used as the template of the second round. After the second PCR reaction, 5 μL amplification product was detected by 1% agarose gel electrophoresis. Nucleotide sequence determination was done by Sangon Biotech Co. Ltd. (Shanghai, China, Beijing Sequencing Department) [20].

The viral gene nucleotide sequences were spliced and corrected using SeqMan II software (DNA Star, Madison, WI, USA). The JEV gene sequences used for phylogenetic analyses were downloaded from GenBank (S1 Table), and the ClustalW multiple sequence alignment was performed using BioEdit (Version 7.0, Hall 1999). Neighbor-joining phylogenetic trees were drawn using MEGA6.0 with 1000 bootstrap replicates [13,21,22].

### Spatiotemporal distribution of JE cases

The data of JE cases from Ningxia in 2018 used in this study were obtained from the National Notifiable Disease Reporting System (NNDRS). The spatiotemporal distribution of JE cases was analyzed using ArcGIS (v.10.0; ESRI, Redlands, CA, USA) [21,22].

## Results

### Laboratory testing of JE case specimens

#### Collection of JE case specimens

Specimens of 70 suspected cases at the acute stage of JEV infection were collected from local hospital in Ningxia, including 45 cases with serum and CSF specimens, 20 cases with serum specimens only, and 5 cases with CSF specimens only. Case specimen information is shown in Table 1.

**Table 1:** Laboratory tests of serum and cerebrospinal specimens of suspected clinical Japanese encephalitis cases, Ningxia, China, 2018.

		IgM			qRT-PCR		
Sample		JEV		WNV	JEV	WNV	Virus isolation
		Beixi	Inbios				
**S**		20/20	20/20	0/20	0/20	0/20	
**S&C**	**S+ C+**	38/38	38/38	3/38	0/38	0/38	
	**S+ C-**	5/5	5/5	4/5	0/5	0/5	
	**S- C+**	0/2	0/2	0/2	1*/2	0/2	1
**Subtotal**		43/45	43/45	7/45	1/45	0/45	1
**C**		4/5	4/5		0/5	0/5	
**Total**		67/70	67/70	7/65	1/70	0/70	1

S: Specimens with serum; C: Specimens with cerebrospinal fluid; S&C: specimens with serum and cerebrospinal fluid.

*This case’s cerebrospinal fluid specimen was positive for the JEV by qRT-PCR and a JEV isolate was obtained (NX1889). S+ C+ indicates that both serum and cerebrospinal fluid specimens were positive; S+ C−indicates that serum was positive but cerebrospinal fluid was negative; S− C+ indicates that serum was negative but cerebrospinal fluid was positive.

#### Detection of JEV and WNV IgM antibodies

Detection of JEV and WNV IgM antibodies was conducted for all collected acute sera. Sixty-seven cases were positive for JEV IgM antibody, and seven were positive for both JEV and WNV IgM antibodies. Using positive viral IgM antibody in the acute phase as the diagnostic criterion [23,24], there were 67/70 cases of JEV infection and 7/70 cases of possible combined JEV and WNV infection (Table 1).

#### Detection of neutralizing antibodies against JEV and WNV

To exclude the possible serological cross-reaction between JEV and WNV, the seven cases positive for JEV and WNV IgM antibody were followed up with collection of convalescent serum. Convalescent serum was obtained from four cases. The detection of neutralizing antibodies against JEV and WNV was carried out in parallel with the acute and convalescent sera of the four cases (Table 2). JEV neutralizing antibody in the acute stage of all four cases was 1:10 or lower, but was 1:40 or 1:80 in the convalescent stage. In all specimens, the WNV neutralizing antibody level was less than 1:10, indicating that these four cases presented with JEV infection rather than WNV.

**Table 2:** Detection of neutralizing antibodies against JEV and WNV.

Number	Gender	Age	Sample	Days post onset	Sample detection							
					IgM			qRT-PCR		NT		
					JEV		WNV	JEV	WNV	JEV	WNV	virus isolation
					Beixi	InBios						
**1**	**female**	65	**S**^**1**^	5	+	+	+	-	-	1:10	<1:10	ND
			**C**	5	+	+	ND	-	-	ND	ND	-
			**S**^**2**^	34	+	+	-	-	-	1:40	<1:10	ND
**2**	**female**	52	**S**^**1**^	8	+	+	+	-	-	<1:10	<1:10	ND
			**S**^**2**^	35	+	+	-	-	-	1: 80	<1:10	ND
**3**	**male**	53	**S**^**1**^	5	+	+	+	-	-	<1:10	<1:10	ND
			**C**	5	+	+	ND	-	-	ND	ND	-
			**S**^**2**^	32	+	+	-	-	-	1:80	<1:10	ND
**4**	**male**	72	**S**^**1**^	5	+	+	+	-	-	<1:10	<1:10	ND
			**S**^**2**^	31	+	+	+	-	-	1:40	<1:10	ND

S1: acute phase serum; S2: convalescent serum; C: cerebrospinal fluid;

NT: neutralizing antibody detection; ND: not done.

#### Viral gene detection (qRT-PCR) and isolation of JEV from CSF specimens

**Fig 1:**
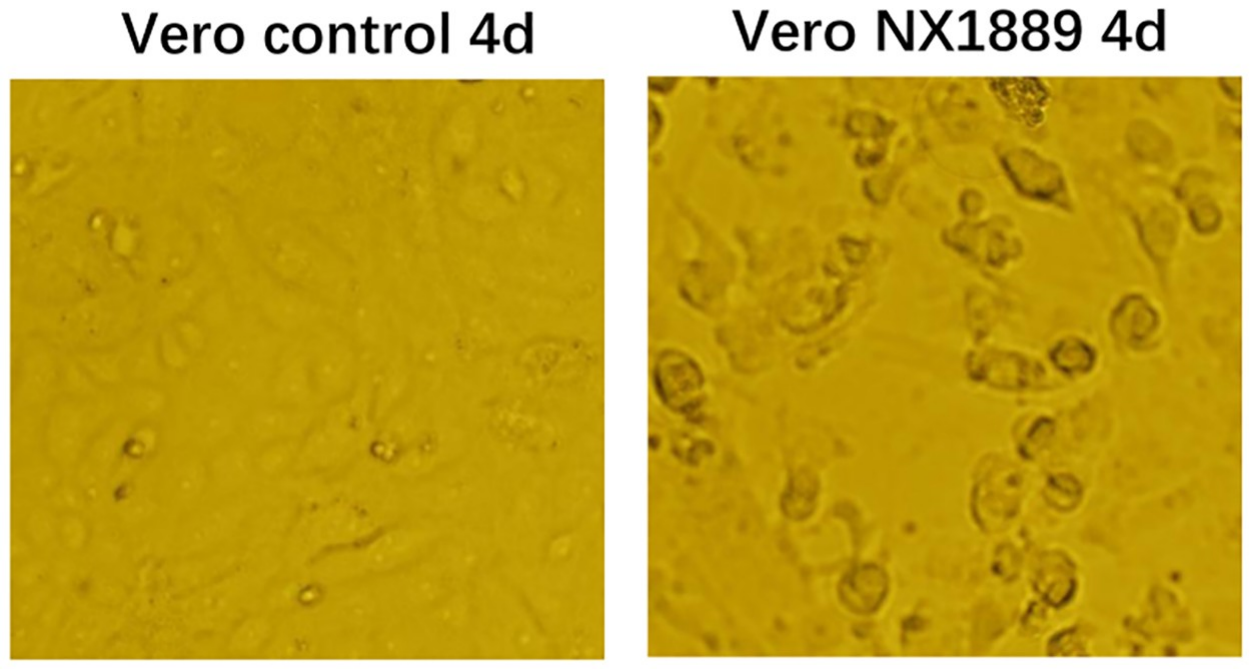
Cytopathic effect (CPE) of virus isolate NX1889 in Vero cells. CPE induced by inoculating CSF specimens from NX1889 cases into Vero cells. Magnification, 200× (10×20). 4 days after inoculation, obvious CPE was observed, which presented as circular shrinkage and shedding of the cell.

qRT-PCR was carried out to determine positive amplification of JEV or WNV in serum and CSF samples in the acute phase. One CSF sample (NX1889) was positive for JEV gene amplification (CT value 30.6) (Table 1). This positive result indicates the presence of JEV, so all the CSF specimen was inoculated into Vero cells for virus isolation. CPE can be serially passaged. Virus gene amplification and nucleotide sequence determination analyses confirmed that the virus isolate was JEV GIb, and was named virus isolate NX1889 (Fig 1).

### General characteristics of the 2018 JE outbreak in Ningxia

#### Epidemiological characteristics

A total of 162 cases of JE with 31 deaths were reported in Ningxia in 2018. Two cases were 0–15 years old, 14 cases were 16–39 years old, and 146 cases were over 40 years old at a proportion of 90.12% (146/162) of the total JE cases in Ningxia in 2018. The male-to-female ratio was 1.31:1 (92:70); 79.01% (128/162) of the cases were farmers, and the rest were students and urban residents. Among the 67 laboratory-confirmed JE cases, two cases were 0–15 years, eight cases were 16–39 years, and 57 cases were over 40 years old (85%; 57/67). The male-to-female ratio was 1.16:1 (36:31); 81% (54/67) of the cases were farmers, followed by 12 urban residents and 1 student.

### Spatiotemporal distribution

#### Spatiotemporal distribution of the 2018 JE outbreak in Ningxia

**Fig 2:**
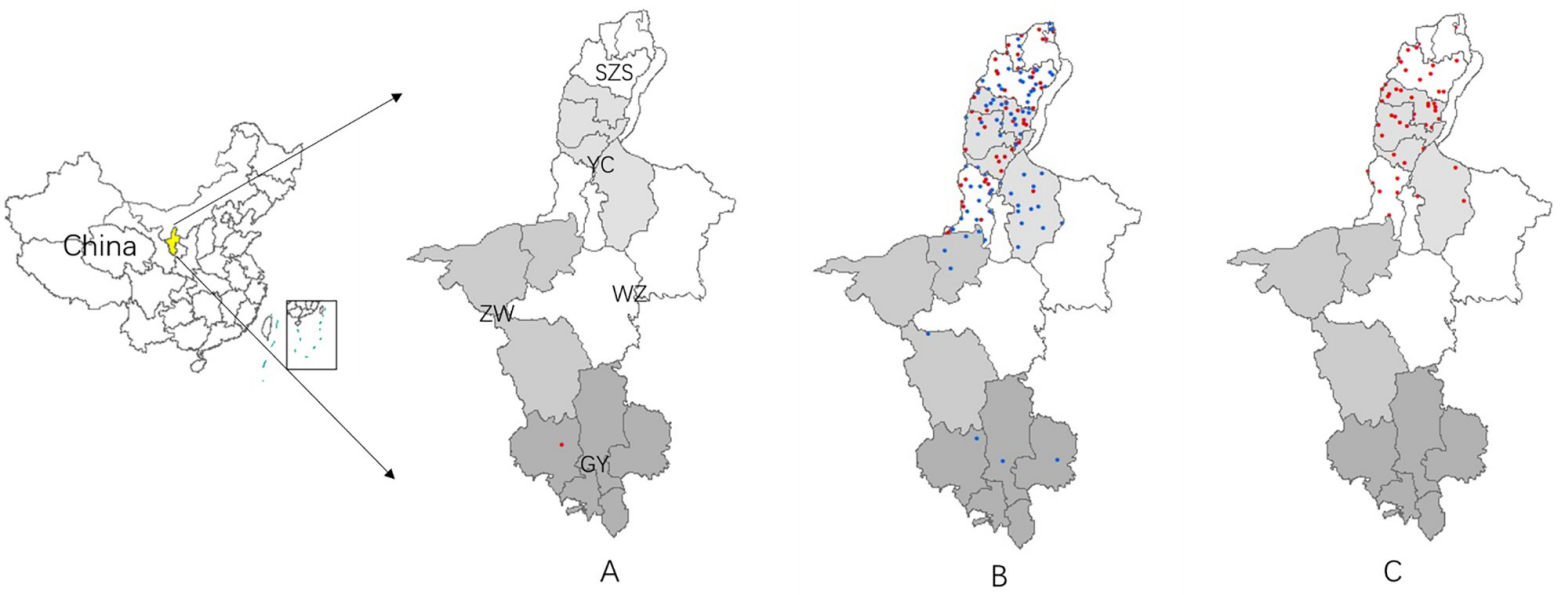
Spatiotemporal distribution of the 2018 JE incidence in Ningxia. The information of JE cases demonstrated on the map were obtained from the National Notifiable Disease Reporting System (NNDRS). The spatiotemporal distribution of JE cases was analyzed using ArcGIS (v.10.0; ESRI, Redlands, CA, USA). Each point represents a case of JE; red points are cases reported (excludes the JE cases diagnosed in this study) in Ningxia in 2018, and blue points are confirmed cases diagnosed in this study. From the south to the north: GY, Guyuan City; ZW, Zhongwei City; WZ, Wuzhong City; YC, Yinchuan City; and SZS, Shizuishan City, same in Fig2(A, B, C). The shading scheme for this map is used to distinguish the five different cities of Ningxia province. Fig2A shows that 1 case was reported in July, Fig2B shows that 113 cases were reported in August, of which 67 were confirmed cases diagnosed in this study, Fig2C shows that 48 cases were reported in September.

Among the 162 cases, the first case occurred in July in the southern part of Guyuan city, and 113 cases were reported in August from five cities, accounting for 70% (113/162) of all the cases in Ningxia in 2018. From the south to the north, reports came from Guyuan (8 cases), Zhongwei (5 cases), Wuzhong (30 cases), Yinchuan (72 cases), and Shizuishan (47 cases). Forty-eight cases were reported in September, and no JE cases were reported after September. In total, the JE cases in Ningxia in 2018 were mainly concentrated in August in Wuzhong City, Yinchuan City, and Shizuishan City, located in the central and northern parts of Ningxia (Fig 2).

#### Spatiotemporal distribution of laboratory-confirmed JE cases

Laboratory tests confirmed 67 cases that occurred in August 2018. These cases were distributed in five cities in Ningxia; from the south to the north: Guyuan (5 cases), Zhongwei (5 cases), Wuzhong (13 cases), Yinchuan (25 cases), and Shizuishan (19 cases). Cases in Wuzhong, Yinchuan, and Shizuishan accounted for 85% (57/67) of the laboratory-confirmed cases (Fig 2). These cases were mainly concentrated in mid to late August; three cases were from 1–10 August, 44 cases were from 11–20 August, and 20 cases were from 21–31 August. A total of 55 JE cases were reported within 10 days from 15–24 August, accounting for 82% (55/67) of the confirmed JE cases in August.

### Investigation of mosquitoes infected with JEV from the local environment

On 20–27 August 2018, mosquito specimens were collected in five villages of Pingluo County, the county with the most JE cases in Shizuishan City. A total of 24,900 mosquitoes were collected belonging to three species: *Culex*. *tritaeniorhynchus* (69.9%; 17,400/24,900), *Culex pipiens pallens* (3800), and *Armigeres obturbans* (3700) (Table 3).

**Table 3:** Mosquito specimen collection and testing by qRT-PCR in Ningxia, 2018.

Collection spot(time)	Mosquito species			Total
	*Culex tritaeniorhynchus*	*Culex pipiens pallens*	*Armigeres obturbans*	
**A(Aug 20th)**	15/310/15500*	0/70/3500	0/70/3500	15/450/22500
**B(Aug 25th)**	0/2/100	0/0/0	0/0/0	0/2/100
**C(Aug 25th)**	0/4/200	0/1/50	0/1/50	0/6/300
**D(Aug 26th)**	1/25/1250	0/4/200	0/3/150	1/32/1600
**E(Aug27th)**	0/7/350	0/1/50	0/0/0	0/8/400
**Total**	16/348/17400	0/76/3800	0/74/3700	16/498/24900

* JEV positive pools /pools tested/number of mosquitoes. The collection locations consisted of five villages in different locations of Pingluo County, Shizuishan City, Ningxia. A, Tongfu Village; B, Gaoren Village; C, Gaozhuang Village; D, Xinfeng Village; and E, Chonggang Village.

All mosquito specimens were divided into 498 pools according to time of collection, location, and mosquito species, and JEV gene detection (qRT-PCR) was performed on each pool. Of 310 pools of *C*. *tritaeniorhynchus*, 15 pools collected on 20 August in Tongfu Village, Pingluo County, tested positive for JEV genes (Table 3, collection site A). In addition, one positive pool was detected from *C*. *tritaeniorhynchus* collected in Xinfeng Village, Pingluo County (Table 3, D collection site) on 26 August. No JEV-positive pools were detected for *C*. *pipiens pallens* (76 pools) or *A*. *obturbans* (74 pools). The supernatants of all 16 pools of JEV-positive mosquito specimens were inoculated into Vero cells and cultured continuously, but no JEV isolate was obtained.

#### Molecular biological characteristics of the 2018 JEV outbreak in Ningxia

Using PCR with JEV-specific primers, positive amplification of the C+PrM and E genes was obtained from virus isolate NX1889. Of the 16 pools of mosquito samples, seven positive amplification and sequence determination results were obtained for the JEV E gene, while nine were positive for the C+PrM gene (Table 4).

**Table 4:** JEV gene detection in cerebrospinal fluid from human JE cases and mosquitoes collected from the local environment in Ningxia, 2018.

Number	qRT-PCR	Amplification of JEV segment		
		PrM+C	E	
			Amplification	Genotype
**Human**				
**NX1889**	+	+	+	GI-b
**Mosquito**				
**NX16**	+	+	+	GI-b
**NX99**	+	-	-	
**NX136**	+	-	-	
**NX141**	+	+	+	GI-b
**NX170**	+	+	-	
**NX176**	+	+	+	GI-b
**NX184**	+	-	-	
**NX251**	+	-	-	
**NX265**	+	-	-	
**NX290**	+	-	-	
**NX311**	+	+	+	GI-b
**NX317**	+	+	+	GI-b
**NX340**	+	+	+	GI-b
**NX404**	+	-	-	
**NX493**	+	+	+	GI-b
**NX193**	+	+	-	
**Positive in total**	17	10	8	8

Table 4 lists the results of JEV genetic testing and sequence determination of virus detected from JE patients (NX1889) and mosquito samples positive for JEV gene amplification. Among them, 16 pools of JEV positive mosquito samples were obtained by qRT-PCR, while 9 and 7 pools were positive of JEV C + PrM and E gene amplification and sequencing, respectively. The results of nucleotide sequence determination and evolution analyses of the 8 E gene positive samples showed that all of them were JEV GIb.

**Fig 3:**
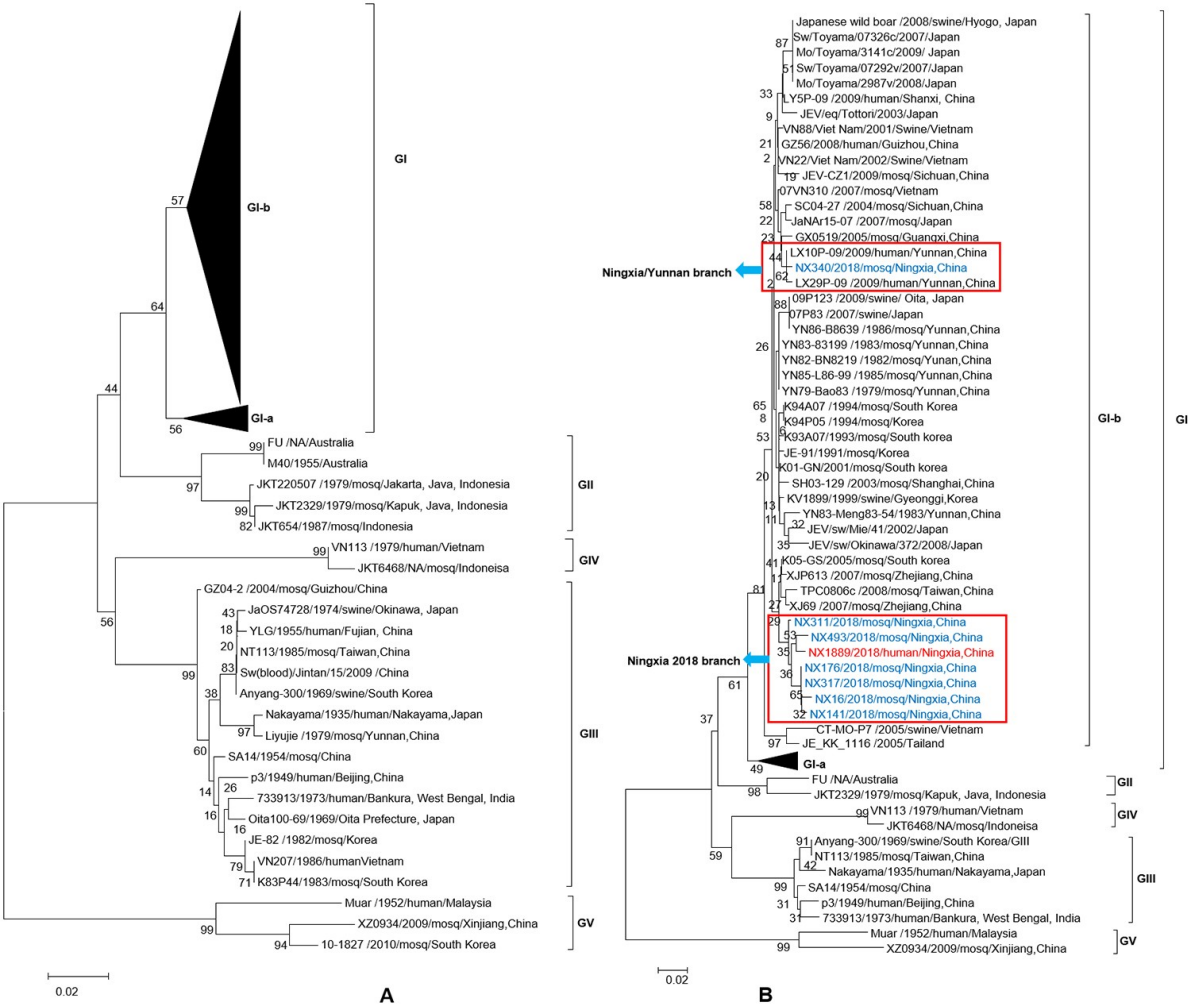
Phylogenetic analyses of the E fragment of JEV from human cases and mosquitoes. Blue indicates JE gene sequences from mosquito specimens of Ningxia in 2018. Red indicates the JEV isolate (NX1889) from cerebrospinal fluid specimens of JE cases. Fig 3A used data set of reported JEV GIb isolates[11] along with JEV GIbs from the 2018 Ningxia outbreak, including seven JEV sequences from mosquitoes and one from human; To visualize the phylogenetic relationship of JEV GIbs from Ningxia, the referenced JEV GIb isolates shown in Fig 3B were selected from Fig 3A and represent different hosts (equid, midge, human, mosquito, pig, and unknown host), different countries (China, Japan, Korea, Korea, Vietnam, Thailand, and Australia), and different times (from the 1970s to 2000s).

Phylogenetic analyses were performed as reported previously [11]to determine the genotypes of the viral sequences obtained from Ningxia in 2018. As shown in Fig 3, JEV was divided into five lineages (five genotypes), of which genotype I could be divided into two clusters, JEV GIa and JEV GIb. JEVs isolated from cases and mosquitoes in Ningxia 2018 belong to the JEV GIb cluster. Fig 3B shows that the viruses isolated in Ningxia in 2018 were grouped into two branches of JEV GIb. One branch is NX340(from mosquito) that forms a separate branch (Ningxia/Yunnan branch) with isolates from two JE cases in Yunnan Province, in 2009. The other branch consists of the remaining JEVs isolated in Ningxia in 2018 that form another separate branch (Ningxia 2018 branch), including isolates from JE cases and mosquitoes from the local environment of Ningxia, 2018. The JEVs of the latter branch were completely independent of the other isolates and became a completely new viral phylogenetic population in the evolution of JEV GIb.

## Discussion

In this study, multiple strains of JEV GIb were obtained from JE cases and *C*. *tritaeniorhynchus* collected from the local environment. Phylogenetic analyses(Fig 3B) showed that JEV GIb was divided into two clades, one of which consisted of two viruses isolated from Vietnam and Thailand in 2005, and the other of which included JEV isolates from mosquitoes and pigs, and cases from China, Japan and Korea, which could be further divided into multiple branches. Among the JEVs GIb that caused the 2018 JE outbreak in Ningxia, there were also two branches, the Ningxia/Yunnan branch, which has circulated in China in the past, and the Ningxia 2018 branch, which has been evolving in the local environment of Ningxia regionally. Thus it can be inferred that JEV GIb was the pathogen that caused the 2018 Ningxia JE outbreak. However, whether the Ningxia 2018 branch could continue to circulate in the local mosquitoes and cause human infection, or appeared only once, as was the case for the two JEV GIb strains isolated from Vietnam and Thailand in 2005. This might become a new issue for monitoring the phylogenetic evolution of JEVs in Ningxia.

In the past two decades, a gradual replacement of JEV GIII by JEV GIb has occurred, JEV GIb is now dominant or co-circulates with JEV GIII in many JE endemic regions [10,25,26]. However, further analyses of the genotype, hosts, and geographical distribution of the isolates revealed that although JEV GIb has become the dominant genotype since 2000, most of the JEVs isolated from JE cases were still JEV GIII (JEV GIb: 54 vs JEV GIII: 175), while most JEV isolates from mosquitoes were JEV GIb (JEV GIb: 278 vs JEV GIII: 197). These findings suggest that although JEV GIb has displaced JEV GIII by achieving a more efficient replication, its host is restricted as evidenced by the fewer number of isolates from cases than JEV GIII as reported [25]. The laboratory results of several JE outbreaks in Asia in recent years also confirm the above analyses. In the JE outbreak in Korea in 2010 (28 cases with 7 deaths), JEV GIb was only detected in local mosquito specimens [27]. In the JE outbreak in India the same year, 13 JEVs were isolated from JE cases, of which 11 were JEV GIII and 2 were JEV GI [28]. The same situation occurred in China, JEV GIII and GI were detected from both cases and local mosquitoes in a JE outbreak with 80 cases and 22 deaths [13]. In other words, although JEV GIb has become the dominant virus, JEV GIII still involved in each reported JE outbreak, In other words, although JEV GIb has become the dominant virus, JEV GIII still involved in each JE outbreak, that is to say, so far, no reports of JE outbreak has been reported with JEV GIb isolated from both collected case samples and local mosquito samples without the involvement of JEV GIII. Therefore, it seems that JEV GIb cannot cause an outbreak of JE in humans alone. However, recent data have shown that the infectivity of JEV GIb in *Culex quinquefasciatus* [29]and *C*. *pipiens* [30] is equivalent to that of JEV GIII and higher than that of JEV GIa. These findings show that JEV GIb has the potential to cause an outbreak, but data to support this is still lacking. In our study of the 2018 Ningxia JE outbreak, only JEV GIb were detected from both JE cases and *C*. *tritaeniorhynchus*, suggesting JEV GIb alone can cause an outbreak. There have been no reports of a JE outbreak caused by JEV GIb alone.

JE is a natural focal disease. The 2018 Ningxia JE outbreak may have been the consequence of many factors such as mosquito vectors, host animals, local immunity, and local climate. The first factor maybe mosquito vectors. Ningxia (also known as the Yellow River Irrigation Area) located in northern China (35° 14’–39° 23’ N, 104° 17’–107°39’ E), is rich in water resources due to the irrigation of the Yellow River, the second largest river in China. As such, rice has gradually become a major crop in the area [31,32]. The cultivation of rice provides a favorable natural environment for the propagation of *C*. *tritaeniorhynchus*, the main vector of JEV [33]. In this study, 17,4000(Table 3) *C*. *tritaeniorhynchus* mosquitoes (dominant, accounting for 70% of mosquitoes locally) were collected from Tongfu Township, Pingluo County, Shizuishan City, where a large number of JE cases occurred, and a large number of JEV-positive mosquitoes were detected (Table 3). Rice cultivation in Ningxia may provide a suitable natural environment for the breeding and reproduction of local *C*. *tritaeniorhynchus*, and may facilitate the spread of JEV. The second factor maybe host animals. With the acceleration of urbanization in China, the number of urban areas in Ningxia is expanding. To supply the growing demand for pork, both the number of pig farmers and the size of pig farms in Ningxia are expanding [34]. Both rice paddies and pig farms exist around the residences of the JE cases reported in Ningxia. There are approximately 13 thousand square meters of rice paddies 20 meters away from the residence of one patient, and two large pig farms with 1000 pigs approximately 300 meters away from the residence of another patient. *C*. *tritaeniorhynchus* and a large number of pigs provide an excellent natural environment for the circulation of JEV in local environment, and both pig farming in courtyards and living near pig farms can increase exposure to JEV [34]. The third factor maybe the low immune barrier. Ningxia has been a low-endemic area of JE, with an incidence of less than 0.1 per 100,000 from 1997 to 2006 [35,36]. In particular, no cases of JE were reported in Ningxia during the 10-year period from 2006 to 2016 [36,37]. Since the Chinese government implemented the EPI administration of the JE vaccine in 2008, children under 15 years old can receive a free JE vaccination; however, the local population born before 2008 was not vaccinated against JE, thus a large number of JEV susceptible individuals may accumulate among local adults (persons born before 2008). The fourth factor maybe climate impact. According to Ningxia meteorological monitoring data, the temperature of Ningxia in the summer of 2018 was the highest since 1961 [38], and the summer temperature was 0.8°C higher than the perennial temperature (an increase in temperature reduces the diurnal temperature difference, which is conducive to mosquito activity and breeding). In addition, the amount of summer precipitation in Ningxia in 2018 increased, with a wide range and high intensity, and the annual precipitation in 2018 was 38% greater than the perennial period. Thus, the special climatic condition in Ningxia in 2018 may also be an important promoter of the local JE epidemic.

As mentioned, JE is a vaccine-preventable disease, and vaccination has almost eliminated JE cases in Japan, South Korea, and Singapore, where JE is traditionally endemic [6]. Between 2008, when China included the JE vaccine in EPI, and 2013, the incidence of JE dropped to 0.56/ 100,000[21,37]. However, the genotype of JEVs in Asia has changed from genotype III to genotype I in recent years [4,9]. In addition, JEV GV remerged in mainland China and South Korea in 2009 after about 60 years of silence[39,40]. Therefore, whether the current JE vaccine (inactivated vaccine and live attenuated vaccine are both from JEV GIII) can effectively protect against the newly emerged JEV genotypes is a concern in the field of virology and public health. In one study, neutralizing antibody titers against JEV GI (GZ56, shown to be JEV Ib in Fig 3), GIII and GV of 26 pairs of serum samples before and after JE vaccination were compared. After vaccination, neutralizing antibodies against JEV GIII showed 100% positive seroconversion rate (SCR), with the highest neutralizing antibody titer reaching 1:1280. The SCR was 96% against GI JEV, with the highest neutralizing antibody titer reaching 1:640. Only a SCR of 35% (9/26) was observed against G5 JEV [17] The above results suggest that the current JE vaccine is less protective against JEV GI and JEV GV infection than against JEV GIII. Furthermore, it is unclear whether people vaccinated with JE vaccine could be infected with JEV Ib or JEV GV. It can be seen that the change of JEV genotype and the emergence of JEV GV pose a challenge to the effectiveness of current JE vaccines in the prevention and control of JE.

As mentioned above, JEV GIb was detected simultaneously in JE case samples (serum and cerebrospinal fluid) and mosquito samples collected in nature in this study, that is, both clinical samples and natural samples suggested that this JE outbreak was caused by JEV GIb infection. However, there are also some shortcomings in this study, for example, this study only obtained one JEV strain (NX1889) from case samples, besides, although a large number of JEV gene-positive were detected in mosquito samples, no JEV isolates were obtained and further analysis in viral molecular biology could not be carried out. In conclusion, to provide more basic etiological data for the relationship between the changes of JEV genotype and local diseases, a long-term detection and monitoring of local JE cases and virus carriage in mosquitoes in Ningxia is needed.

### Conclusion

This study reported an outbreak of JE caused by JEV GIb in northeast Asia using evidence from both laboratory and field data, which showed that JEV GIb can separately cause an outbreak. (the number of JEV obtained from patient samples in this study was limited, and continuous surveillance is also needed). The molecular epidemiology of JEV GIb and epidemiology characteristic of this outbreak can be a reference for JE control and prevention of northern Asia. The outbreak shows that attention should be paid to the circulation of JEV GIb and its potential of causing an outbreak. In addition, JEV is mainly transmitted by mosquitoes (*Culex tritaeniorhynchus*), regardless of the genotype, and the clinical symptoms caused by different genotypes of JEV infection are also the same. Therefore, it is recommended that molecular biological test of JEV genotype and sub-genotype be performed along with routine detection of JEV IgM antibody and viral genes in patients infected by JEV, to clarify the threat of JEV GIb to public health.

## Polish statement

The English in this document has been checked by at least two professional editors, both native speakers of English. For a certificate, please see:

http://www.textcheck.com/certificate/CJO6R8

## Supporting information

S1 Table Strains of Japanese encephalitis virus used in this study. (DOCX)
