# Supplementary material for: An outbreak of Japanese encephalitis caused by genotype Ib Japanese encephalitis virus in China, 2018: A laboratory and field investigation
Source: PLoS Negl Trop Dis. 2020 May 26;14(5):e0008312. doi: 10.1371/journal.pntd.0008312 (PMC7274457; doi:10.1371/journal.pntd.0008312)
Supplement: S1 Table — (DOCX) [file pntd.0008312.s001.docx]

Supplementary Table :Strains of Japanese encephalitis virus used in this study

| **Strain** | **Genotype** | **Country** | **Year** | **Host** | **GenBank accession no.** |
| --- | --- | --- | --- | --- | --- |
| KE-93-83 | GI-a | Thailand | 1983 | Mosquito | KF192510 |
| TS00 | GI-a | Badu Island, Australia | 2000 | Swine | EF434785 |
| JE_RT_36 | GI-a | Ratchaburi, Thailand | 2003 | Mosquito | DQ087975 |
| JE_CP_49 | GI-a | Chumphon, Thailand | 2004 | Swine | DQ087974 |
| JE_CP_67 | GI-a | Chumphon, Thailand | 2004 | Swine | DQ087972 |
| JE_KK_80 | GI-a | Khon Khen, Thailand | 2004 | Unknown | DQ111784 |
| JE_KK_82 | GI-a | Khon Khen, Thailand | 2004 | Unknown | DQ111785 |
| JE_KK_R83 | GI-a | Khon Khen, Thailand | 2004 | Unknown | DQ111787 |
| JE_KK_R87 | GI-a | Khon Khen, Thailand | 2004 | Unknown | DQ111788 |
| JE_KK_R88 | GI-a | Khon Khen, Thailand | 2004 | Unknown | DQ111786 |
| JE_PK52 | GI-a | Phuket, Thailand | 2004 | Unknown | DQ84229 |
| JE_CM_1196 | GI-a | Chiang Mai, Thailand | 2005 | Unknown | DQ238602 |
| JE_KK_577 | GI-a | Khon Khen, Thailand | 2005 | Unknown | DQ238601 |
| JE_KK_580 | GI-a | Khon Khen, Thailand | 2005 | Unknown | DQ238600 |
| YN79-Bao83 | GI-b | Yunnan, China | 1979 | Mosquito | DQ404128 |
| YN82-BN8219 | GI-b | Yunnan, China | 1982 | Mosquito | DQ404129 |
| YN83-83199 | GI-b | Yunnan, China | 1983 | Mosquito | DQ404131 |
| YN83-Meng83-54 | GI-b | Yunnan, China | 1983 | Midge | DQ404130 |
| YN85-L86-99 | GI-b | Yunnan, China | 1985 | Mosquito | DQ404132 |
| YN86-86266 | GI-b | Yunnan, China | 1986 | Unknown | DQ404134 |
| YN86-B8639 | GI-b | Yunnan, China | 1986 | Mosquito | DQ404133 |
| JE-91 | GI-b | Korea | 1991 | Mosquito | GQ415355 |
| K93A07 | GI-b | South Korea | 1993 | Mosquito | FJ938230 |
| K94P05 | GI-b | Korea | 1994 | Mosquito | AF045551 |
| K94A07 | GI-b | South Korea | 1994 | Mosquito | FJ938216 |
| JaTAn 1/94 | GI-b | Tokyo, Japan | 1994 | Swine | AB237171 |
| 95-167 | GI-b | Japan | 1995 | Swine | AY377579 |
| 95-91 | GI-b | Japan | 1995 | Swine | AY377578 |
| 95P99 | GI-b | Oita, Japan | 1995 | Swine | FJ943471 |
| K95A07 | GI-b | South Korea | 1995 | Mosquito | FJ938218 |
| K96A07 | GI-b | South Korea | 1996 | Mosquito | FJ938219 |
| 97P82 | GI-b | Oita, Japan | 1997 | Swine | FJ943472 |
| Ishikawa | GI-b | Ishikawa, Japan | 1998 | Mosquito | AB051292 |
| JEV/wb/Okinawa/1/1998 | GI-b | Okinawa, Japan | 1998 | Swine | AB306941 |
| 99P103 | GI-b | Oita, Japan | 1999 | Swine | FJ943473 |
| 99P104 | GI-b | Oita, Japan | 1999 | Swine | FJ943474 |
| KV1899 | GI-b | South Korea | 1999 | Swine | AF474075 |
| SH-101 | GI-b | Shanghai, China | 2001 | Mosquito | AY555761 |
| SH-53 | GI-b | Shanghai, China | 2001 | Mosquito | AY555757 |
| SH-80 | GI-b | Shanghai, China | 2001 | Mosquito | AY243841 |
| SH-81 | GI-b | Shanghai, China | 2001 | Mosquito | AY555758 |
| SH-83 | GI-b | Shanghai, China | 2001 | Mosquito | AY555759 |
| SH-90 | GI-b | Shanghai, China | 2001 | Mosquito | AY243835 |
| SH-96 | GI-b | Shanghai, China | 2001 | Mosquito | AY555760 |
| K01-GN | GI-b | South Korea | 2001 | Mosquito | FJ938220 |
| K01-JB | GI-b | South Korea | 2001 | Mosquito | FJ938221 |
| K01-JN | GI-b | South Korea | 2001 | Mosquito | FJ938222 |
| VN88 | GI-b | Vietnam | 2001 | Swine | AY376464 |
| JEV/sw/Chiba/88/2002 | GI-b | Chiba, Japan | 2002 | Swine | AB112705 |
| JEV/sw/Hiroshima/25/2002 | GI-b | Hiroshima, Japan | 2002 | Swine | AB231465 |
| JaNAr0102 | GI-b | Japan | 2002 | Mosquito | AY377577 |
| JEV/sw/Kagawa/24/2002 | GI-b | Kagawa, Japan | 2002 | Swine | AB112706 |
| JEV/sw/Kagawa/27/2002 | GI-b | Kagawa, Japan | 2002 | Swine | AB112707 |
| LN02-102 | GI-b | Liaoning, China | 2002 | Mosquito | DQ404085 |
| LN02-104 | GI-b | Liaoning, China | 2002 | Mosquito | DQ404086 |
| JEV/sw/Mie/41/2002 | GI-b | Mie, Japan | 2002 | Swine | AB112709 |
| JEV/sw/Shizuoka/33/2002 | GI-b | Shizuoka, Japan | 2002 | Swine | AB112703 |
| JEV/sw/Shizuoka/39/2002 | GI-b | Shizuoka, Japan | 2002 | Swine | AB112704 |
| VN105 | GI-b | Vietnam | 2002 | Mosquito | AY376468 |
| VN22 | GI-b | Vietnam | 2002 | Swine | AY376465 |
| VN34 | GI-b | Vietnam | 2002 | Mosquito | AY376466 |
| VN78 | GI-b | Vietnam | 2002 | Mosquito | AY376467 |
| 03P113 | GI-b | Oita, Japan | 2003 | Swine | FJ943475 |
| 03P120 | GI-b | Oita, Japan | 2003 | Swine | FJ943476 |
| 03P126 | GI-b | Oita, Japan | 2003 | Swine | FJ943477 |
| 03P145 | GI-b | Oita, Japan | 2003 | Swine | FJ943478 |
| 03P189 | GI-b | Oita, Japan | 2003 | Swine | FJ943479 |
| JEV/sw/Okinawa/285/2003 | GI-b | Okinawa, Japan | 2003 | Swine | AB238693 |
| SH03-103 | GI-b | Shanghai, China | 2003 | Mosquito | DQ404096 |
| SH03-105 | GI-b | Shanghai, China | 2003 | Mosquito | DQ404097 |
| SH03-109 | GI-b | Shanghai, China | 2003 | Mosquito | DQ404098 |
| SH03-115 | GI-b | Shanghai, China | 2003 | Mosquito | DQ404099 |
| SH03-124 | GI-b | Shanghai, China | 2003 | Mosquito | DQ404100 |
| SH03-127 | GI-b | Shanghai, China | 2003 | Mosquito | DQ404101 |
| SH03-128 | GI-b | Shanghai, China | 2003 | Mosquito | DQ404102 |
| SH03-129 | GI-b | Shanghai, China | 2003 | Mosquito | DQ404103 |
| SH03-130 | GI-b | Shanghai, China | 2003 | Mosquito | DQ404104 |
| JEV/eq/Tottori/2003 | GI-b | Tottori, Japan | 2003 | Equid | AB213007 |
| HN04-11 | GI-b | Henan, China | 2004 | Mosquito | DQ404087 |
| HN04-21 | GI-b | Henan, China | 2004 | Mosquito | DQ404088 |
| HN04-40 | GI-b | Henan, China | 2004 | Mosquito | DQ404089 |
| JaNAr07-04 | GI-b | Isahaya, Nagasaki Prefecture, Japan | 2004 | Mosquito | FJ185144 |
| JaNAr10-04 | GI-b | Isahaya, Nagasaki Prefecture, Japan | 2004 | Mosquito | FJ185145 |
| JaNAr13-04 | GI-b | Isahaya, Nagasaki Prefecture, Japan | 2004 | Mosquito | FJ185146 |
| JaNAr31-04 | GI-b | Isahaya, Nagasaki Prefecture, Japan | 2004 | Mosquito | FJ185150 |
| JaNAr32-04 | GI-b | Isahaya, Nagasaki Prefecture, Japan | 2004 | Mosquito | FJ185151 |
| JaNAr38-04 | GI-b | Isahaya, Nagasaki Prefecture, Japan | 2004 | Mosquito | FJ185152 |
| JEV/sw/Kagawa/35/2004 | GI-b | Kagawa, Japan | 2004 | Swine | AB231464 |
| JEV/sw/Mie/34/2004 | GI-b | Mie, Japan | 2004 | Swine | AB231462 |
| JEV/sw/Mie/40/2004 | GI-b | Mie, Japan | 2004 | Swine | AB231463 |
| SC04-12 | GI-b | Sichuan, China | 2004 | Mosquito | DQ404090 |
| SC04-15 | GI-b | Sichuan, China | 2004 | Mosquito | DQ404091 |
| SC04-16 | GI-b | Sichuan, China | 2004 | Mosquito | DQ404092 |
| SC04-17 | GI-b | Sichuan, China | 2004 | Mosquito | DQ404093 |
| SC04-25 | GI-b | Sichuan, China | 2004 | Mosquito | DQ404094 |
| SC04-27 | GI-b | Sichuan, China | 2004 | Mosquito | DQ404095 |
| GX0519 | GI-b | Guangxi, China | 2005 | Mosquito | FJ161967 |
| GX0523 | GI-b | Guangxi, China | 2005 | Mosquito | FJ161968 |
| GX0558 | GI-b | Guangxi, China | 2005 | Mosquito | FJ161969 |
| JE_KK_1116 | GI-b | Khon Khen, Thailand | 2005 | Unknown | DQ343290 |
| 05P75 | GI-b | Oita, Japan | 2005 | Swine | FJ943480 |
| SH05-24 | GI-b | Shanghai, China | 2005 | Mosquito | DQ404108 |
| K05-GS | GI-b | South Korea | 2005 | Mosquito | FJ938223 |
| Mo/Toyama/1089c/2005 | GI-b | Toyama, Japan | 2005 | Mosquito | AB538603 |
| Mo/Toyama/1089v/2005 | GI-b | Toyama, Japan | 2005 | Mosquito | AB538604 |
| Mo/Toyama/1148c/2005 | GI-b | Toyama, Japan | 2005 | Mosquito | AB538605 |
| Mo/Toyama/1148v/2005 | GI-b | Toyama, Japan | 2005 | Mosquito | AB538606 |
| Mo/Toyama/1149c/2005 | GI-b | Toyama, Japan | 2005 | Mosquito | AB538607 |
| Mo/Toyama/1155c/2005 | GI-b | Toyama, Japan | 2005 | Mosquito | AB538608 |
| Mo/Toyama/1155v/2005 | GI-b | Toyama, Japan | 2005 | Mosquito | AB538609 |
| Mo/Toyama/1157c/2005 | GI-b | Toyama, Japan | 2005 | Mosquito | AB538610 |
| Mo/Toyama/1158c/2005 | GI-b | Toyama, Japan | 2005 | Mosquito | AB538611 |
| Mo/Toyama/1158v/2005 | GI-b | Toyama, Japan | 2005 | Mosquito | AB538612 |
| Mo/Toyama/1160c/2005 | GI-b | Toyama, Japan | 2005 | Mosquito | AB538613 |
| Mo/Toyama/1161c/2005 | GI-b | Toyama, Japan | 2005 | Mosquito | AB538614 |
| Mo/Toyama/1161v/2005 | GI-b | Toyama, Japan | 2005 | Mosquito | AB538615 |
| Mo/Toyama/1222c/2005 | GI-b | Toyama, Japan | 2005 | Mosquito | AB538616 |
| Mo/Toyama/1222v/2005 | GI-b | Toyama, Japan | 2005 | Mosquito | AB538617 |
| Mo/Toyama/1256c/2005 | GI-b | Toyama, Japan | 2005 | Mosquito | AB538618 |
| Mo/Toyama/1256v/2005 | GI-b | Toyama, Japan | 2005 | Mosquito | AB538619 |
| Sw/Toyama/05197v/2005 | GI-b | Toyama, Japan | 2005 | Swine | AB538823 |
| Sw/Toyama/05231v/2005 | GI-b | Toyama, Japan | 2005 | Swine | AB538824 |
| Mo/Toyama/1018c/2005 | GI-b | Toyama, Japan | 2005 | Mosquito | AB538601 |
| Mo/Toyama/1018v/2005 | GI-b | Toyama, Japan | 2005 | Mosquito | AB538602 |
| CT-MO-P7 | GI-b | Vietnam | 2005 | Swine | HQ009266 |
| LA_H06-05 | GI-b | Vietnam | 2005 | Mosquito | FJ185153 |
| LA_H07-05 | GI-b | Vietnam | 2005 | Mosquito | FJ185154 |
| LA-H-5330 | GI-b | Vietnam | 2005 | Swine | HQ009265 |
| LAH_2079-05 | GI-b | Vietnam | 2005 | Mosquito | FJ185155 |
| 06P152 | GI-b | Oita, Japan | 2006 | Swine | FJ943481 |
| 06P183 | GI-b | Oita, Japan | 2006 | Swine | FJ943483 |
| 06P212 | GI-b | Oita, Japan | 2006 | Swine | FJ943484 |
| HEN0701 | GI-b | Henan, China | 2007 | Swine | FJ156730 |
| JaNAr06-07 | GI-b | Isahaya, Nagasaki Prefecture, Japan | 2007 | Mosquito | FJ185143 |
| JaNAr14-07 | GI-b | Isahaya, Nagasaki Prefecture, Japan | 2007 | Mosquito | FJ185147 |
| JaNAr15-07 | GI-b | Isahaya, Nagasaki Prefecture, Japan | 2007 | Mosquito | FJ185148 |
| JaNAr17-07 | GI-b | Isahaya, Nagasaki Prefecture, Japan | 2007 | Mosquito | FJ185149 |
| 07P127 | GI-b | Oita, Japan | 2007 | Swine | FJ943487 |
| 07P83 | GI-b | Oita, Japan | 2007 | Swine | FJ943485 |
| 07P90 | GI-b | Oita, Japan | 2007 | Swine | FJ943486 |
| Mo/Toyama/2347c/2007 | GI-b | Toyama, Japan | 2007 | Mosquito | AB538658 |
| Mo/Toyama/2441c/2007 | GI-b | Toyama, Japan | 2007 | Mosquito | AB538659 |
| Mo/Toyama/2462c/2007 | GI-b | Toyama, Japan | 2007 | Mosquito | AB538660 |
| Mo/Toyama/2506c/2007 | GI-b | Toyama, Japan | 2007 | Mosquito | AB538661 |
| Mo/Toyama/2507c/2007 | GI-b | Toyama, Japan | 2007 | Mosquito | AB538662 |
| Mo/Toyama/2513c/2007 | GI-b | Toyama, Japan | 2007 | Mosquito | AB538663 |
| Mo/Toyama/2513v/2007 | GI-b | Toyama, Japan | 2007 | Mosquito | AB538664 |
| Mo/Toyama/2554c/2007 | GI-b | Toyama, Japan | 2007 | Mosquito | AB538665 |
| Mo/Toyama/2554v/2007 | GI-b | Toyama, Japan | 2007 | Mosquito | AB538666 |
| Mo/Toyama/2556c/2007 | GI-b | Toyama, Japan | 2007 | Mosquito | AB538667 |
| Mo/Toyama/2556v/2007 | GI-b | Toyama, Japan | 2007 | Mosquito | AB538668 |
| Mo/Toyama/2567c/2007 | GI-b | Toyama, Japan | 2007 | Mosquito | AB538669 |
| Mo/Toyama/2569c/2007 | GI-b | Toyama, Japan | 2007 | Mosquito | AB538670 |
| Mo/Toyama/2569v/2007 | GI-b | Toyama, Japan | 2007 | Mosquito | AB538671 |
| Sw/Toyama/07232c/2007 | GI-b | Toyama, Japan | 2007 | Swine | AB538825 |
| Sw/Toyama/07234c/2007 | GI-b | Toyama, Japan | 2007 | Swine | AB538826 |
| Sw/Toyama/07240c/2007 | GI-b | Toyama, Japan | 2007 | Swine | AB538827 |
| Sw/Toyama/07292c/2007 | GI-b | Toyama, Japan | 2007 | Swine | AB538828 |
| Sw/Toyama/07292v/2007 | GI-b | Toyama, Japan | 2007 | Swine | AB538829 |
| Sw/Toyama/07296c/2007 | GI-b | Toyama, Japan | 2007 | Swine | AB538830 |
| Sw/Toyama/07326c/2007 | GI-b | Toyama, Japan | 2007 | Swine | AB538831 |
| 07VN310 | GI-b | Vietnam | 2007 | Mosquito | HM228922 |
| 07VN311 | GI-b | Vietnam | 2007 | Mosquito | HM228923 |
| XJ69 | GI-b | Zhejiang, China | 2007 | Mosquito | EU258742 |
| XJP613 | GI-b | Zhejiang, China | 2007 | Mosquito | EU258741 |
| Japanese wild boar | GI-b | Hyogo, Nishinomiya Prefecture, Japan | 2008 | Swine | AB481224 |
| 08P37 | GI-b | Oita, Japan | 2008 | Swine | FJ943488 |
| 08P38 | GI-b | Oita, Japan | 2008 | Swine | FJ943489 |
| 08P42 | GI-b | Oita, Japan | 2008 | Swine | FJ943490 |
| 08P48 | GI-b | Oita, Japan | 2008 | Swine | FJ943491 |
| 08P49 | GI-b | Oita, Japan | 2008 | Swine | FJ943492 |
| 08P54 | GI-b | Oita, Japan | 2008 | Swine | FJ943493 |
| 08P62 | GI-b | Oita, Japan | 2008 | Swine | FJ943494 |
| JEV/sw/Okinawa/154/2008 | GI-b | Okinawa, Japan | 2008 | Swine | AB471666 |
| JEV/sw/Okinawa/254/2008 | GI-b | Okinawa, Japan | 2008 | Swine | AB471667 |
| JEV/sw/Okinawa/372/2008 | GI-b | Okinawa, Japan | 2008 | Swine | AB471668 |
| JEV/sw/Okinawa/377/2008 | GI-b | Okinawa, Japan | 2008 | Swine | AB471669 |
| JEV/sw/Okinawa/402/2008 | GI-b | Okinawa, Japan | 2008 | Swine | AB471670 |
| TPC0806c | GI-b | Taipei County, Taiwan | 2008 | Mosquito | GQ260635 |
| Mo/Toyama/2757c/2008 | GI-b | Toyama, Japan | 2008 | Mosquito | AB538700 |
| Mo/Toyama/2759c/2008 | GI-b | Toyama, Japan | 2008 | Mosquito | AB538701 |
| Mo/Toyama/2794c/2008 | GI-b | Toyama, Japan | 2008 | Mosquito | AB538702 |
| Mo/Toyama/2794v/2008 | GI-b | Toyama, Japan | 2008 | Mosquito | AB538703 |
| Mo/Toyama/2795c/2008 | GI-b | Toyama, Japan | 2008 | Mosquito | AB538704 |
| Mo/Toyama/2795v/2008 | GI-b | Toyama, Japan | 2008 | Mosquito | AB538705 |
| Mo/Toyama/2805c/2008 | GI-b | Toyama, Japan | 2008 | Mosquito | AB538706 |
| Mo/Toyama/2805v/2008 | GI-b | Toyama, Japan | 2008 | Mosquito | AB538707 |
| Mo/Toyama/2808c/2008 | GI-b | Toyama, Japan | 2008 | Mosquito | AB538708 |
| Mo/Toyama/2821c/2008 | GI-b | Toyama, Japan | 2008 | Mosquito | AB538709 |
| Mo/Toyama/2842c/2008 | GI-b | Toyama, Japan | 2008 | Mosquito | AB538710 |
| Mo/Toyama/2842v/2008 | GI-b | Toyama, Japan | 2008 | Mosquito | AB538711 |
| Mo/Toyama/2853c/2008 | GI-b | Toyama, Japan | 2008 | Mosquito | AB538712 |
| Mo/Toyama/2853v/2008 | GI-b | Toyama, Japan | 2008 | Mosquito | AB538713 |
| Mo/Toyama/2886c/2008 | GI-b | Toyama, Japan | 2008 | Mosquito | AB538714 |
| Mo/Toyama/2886v/2008 | GI-b | Toyama, Japan | 2008 | Mosquito | AB538715 |
| Mo/Toyama/2888c/2008 | GI-b | Toyama, Japan | 2008 | Mosquito | AB538716 |
| Mo/Toyama/2895c/2008 | GI-b | Toyama, Japan | 2008 | Mosquito | AB538717 |
| Mo/Toyama/2905c/2008 | GI-b | Toyama, Japan | 2008 | Mosquito | AB538718 |
| Mo/Toyama/2906c/2008 | GI-b | Toyama, Japan | 2008 | Mosquito | AB538719 |
| Mo/Toyama/2909c/2008 | GI-b | Toyama, Japan | 2008 | Mosquito | AB538720 |
| Mo/Toyama/2910c/2008 | GI-b | Toyama, Japan | 2008 | Mosquito | AB538721 |
| Mo/Toyama/2915c/2008 | GI-b | Toyama, Japan | 2008 | Mosquito | AB538722 |
| Mo/Toyama/2917c/2008 | GI-b | Toyama, Japan | 2008 | Mosquito | AB538723 |
| Mo/Toyama/2918c/2008 | GI-b | Toyama, Japan | 2008 | Mosquito | AB538724 |
| Mo/Toyama/2929c/2008 | GI-b | Toyama, Japan | 2008 | Mosquito | AB538725 |
| Mo/Toyama/2929v/2008 | GI-b | Toyama, Japan | 2008 | Mosquito | AB538726 |
| Mo/Toyama/2967c/2008 | GI-b | Toyama, Japan | 2008 | Mosquito | AB538727 |
| Mo/Toyama/2967v/2008 | GI-b | Toyama, Japan | 2008 | Mosquito | AB538728 |
| Mo/Toyama/2976c/2008 | GI-b | Toyama, Japan | 2008 | Mosquito | AB538729 |
| Mo/Toyama/2976v/2008 | GI-b | Toyama, Japan | 2008 | Mosquito | AB538730 |
| Mo/Toyama/2977c/2008 | GI-b | Toyama, Japan | 2008 | Mosquito | AB538731 |
| Mo/Toyama/2977v/2008 | GI-b | Toyama, Japan | 2008 | Mosquito | AB538732 |
| Mo/Toyama/2984c/2008 | GI-b | Toyama, Japan | 2008 | Mosquito | AB538733 |
| Mo/Toyama/2984v/2008 | GI-b | Toyama, Japan | 2008 | Mosquito | AB538734 |
| Mo/Toyama/2985c/2008 | GI-b | Toyama, Japan | 2008 | Mosquito | AB538735 |
| Mo/Toyama/2985v/2008 | GI-b | Toyama, Japan | 2008 | Mosquito | AB538736 |
| Mo/Toyama/2986c/2008 | GI-b | Toyama, Japan | 2008 | Mosquito | AB538737 |
| Mo/Toyama/2986v/2008 | GI-b | Toyama, Japan | 2008 | Mosquito | AB538738 |
| Mo/Toyama/2987c/2008 | GI-b | Toyama, Japan | 2008 | Mosquito | AB538739 |
| Mo/Toyama/2987v/2008 | GI-b | Toyama, Japan | 2008 | Mosquito | AB538740 |
| Sw/Toyama/08253c/2008 | GI-b | Toyama, Japan | 2008 | Swine | AB538832 |
| YL0806f | GI-b | Yilan County, Taiwan | 2008 | Mosquito | GQ260633 |
| FQ24M-08 | GI-b | Yunnan, China | 2008 | Mosquito | HM204531 |
| XP174M-08 | GI-b | Yunnan, China | 2008 | Mosquito | HM204527 |
| 09P123 | GI-b | Oita, Japan | 2009 | Swine | GU108334 |
| 09P141 | GI-b | Oita, Japan | 2009 | Swine | GU108335 |
| LY5P-09 | GI-b | Shanxi, China | 2009 | Human | HM204530 |
| JEV-CZ1 | GI-b | Sichuan, China | 2009 | Mosquito | HM234673 |
| Mo/Toyama/3133c/2009 | GI-b | Toyama, Japan | 2009 | Mosquito | AB543738 |
| Mo/Toyama/3140c/2009 | GI-b | Toyama, Japan | 2009 | Mosquito | AB543739 |
| Mo/Toyama/3141c/2009 | GI-b | Toyama, Japan | 2009 | Mosquito | AB543740 |
| LX10P-09 | GI-b | Yunnan, China | 2009 | Human | HM204528 |
| LX29P-09 | GI-b | Yunnan, China | 2009 | Human | HM204529 |
| NX1889 | GI-b | Ningxia,China | 2018 | Human | MT134112 |
| NX16 | GI-b | Ningxia,China | 2018 | Mosquito |  |
| NX141 | GI-b | Ningxia,China | 2018 | Mosquito |  |
| NX176 | GI-b | Ningxia,China | 2018 | Mosquito |  |
| NX311 | GI-b | Ningxia,China | 2018 | Mosquito |  |
| NX317 | GI-b | Ningxia,China | 2018 | Mosquito |  |
| NX340 | GI-b | Ningxia,China | 2018 | Mosquito |  |
| NX493 | GI-b | Ningxia,China | 2018 | Mosquito |  |
| FU | GII | Austrilia | 1995 | Human | AF217620 |
| M40 | GII | Austrilia | 1995 | Unknown | L47350 |
| JKT220507 | GII | Jakarta, Java, Indonesia | 1979 | Mosquito | JQ429291 |
| JKT2329 | GII | Kapuk, Java, Indonesia | 1979 | Mosquito | JQ429298 |
| JKT654 | GII | Indonesia | 1978 | Mosquito | HQ223287 |
| P3 | GIII | China | 1949 | Human | U47032.1 |
| Nakayama | GIII | Japan | 1935 | Human | EF688640 |
| Anyang-300 | GIII | South Korea | 1969 | Swine | KT447437 |
| SA14 | GIII | China | 1954 | Mosquito | U14163 |
| Liyujie | GIII | Yunnan, China | 1979 | Human | FJ185039 |
| JaOS74728 | GIII | Okinawa, Japan | 1974 | Swine | AB569992 |
| YLG | GIII | Fujian, China | 1955 | Human | AY243837 |
| NT113 | GIII | Tiachung County, Taiwan | 1985 | Mosquito | U44968 |
| GZ04-2 | GIII | Guizhou, China | 2004 | Mosquito | DQ404109 |
| Sw(blood)/Jintan/15/2009 | GIII | Jintan, China | 2009 | Swine | GU253953 |
| 733913 | GIII | Bankura, West Bengal, India | 1973 | Human | EU372660 |
| Oita100-69 | GIII | Oita Prefecture, Japan | 1969 | Unknown | AB028269 |
| JE-82 | GIII | Korea | 1982 | Mosquito | GQ415347 |
| VN207 | GIII | Vietnam | 1986 | Human | AY376461 |
| K83p44 | GIII | South Korea | 1983 | Mosquito | FJ938232 |
| VN 113 | GIV | Vietnam | 1979 | Human | KU705228 |
| JKT6468 | GIV | Indonesia | 1981 | Mosquito | AY184212 |
| Muar | GV | Malaysia | 1952 | Human | HM596272 |
| XZ0934 | GV | China | 2009 | Mosquito | JF915894 |
| 10-1827 | GV | South Korea | 2010 | Mosquito | JN587258 |
